# Detection of Divergent Highly Pathogenic Avian Influenza A(H5N1) Clade 2.3.4.4b Virus, São Paulo, Brazil, 2025

**DOI:** 10.3201/eid3210.260723

**Published:** 2026-10

**Authors:** Jansen de Araujo, Luciano M. Thomazelli, Thomas P. Fabrizio, Gustavo O. Fenner, Erick G. Dorlass, Ana K.A. Eisen, Viviane F. Botosso, Marcello S. Nardi, Caroline C. Aires, Sergio M.N. Teixeira, Roberta M. de Azevedo, Mayra H. Frediani, Tatiana Ometto, Mário G.L. Barboza, Edmarcia E. de Souza, Carsten Wrenger, Richard J. Webby, Edison L. Durigon

**Affiliations:** University of São Paulo, São Paulo, Brazil (J. de Araujo, L.M. Thomazelli, G.O. Fenner, E.G. Dorlass, A.K.A. Eisen, M.G.L. Barboza, E.E. de Souza, C. Wrenger, E.L. Durigon); St. Jude Children’s Research Hospital, Memphis, Tennessee, USA (T.P. Fabrizio, R.J. Webby); Hospital Israelita Albert Einstein, São Paulo (E.G. Dorlass); Instituto Butantan, São Paulo (V.F. Botosso); Centro de Manejo e Conservação de Animais Silvestres, São Paulo (M.S. Nardi, C.C. Aires, S.M.N. Teixeira, R.M. de Azevedo, M.H. Frediani); Centro Nacional de Pesquisa em Energia e Materiais, Campinas, Brazil (T. Ometto); Institut Pasteur de São Paulo, São Paulo (C. Wrenger, E.L. Durigon).

**Keywords:** Influenza, viruses, viral, highly pathogenic avian influenza, H5N1, genomic surveillance, Brazil

## Abstract

In 2025, we detected highly pathogenic avian influenza H5N1 virus in dead waterfowl at Ibirapuera Park, São Paulo, Brazil. Genomic characterization indicated a reassortant virus that emerged from locally circulating low pathogenicity avian influenza viruses and highly pathogenic North American lineages. Our results highlight cross-species transmission risk and underscore the need for enhanced surveillance.

Avian influenza virus (AIV) surveillance in South America has been limited compared with other regions. Until 2022, only 1 highly pathogenic avian influenza (HPAI) virus outbreak was reported in South America, a 2002 H7N3 event in Chile, which emerged locally (*1*). Most viruses detected before 2022 were low pathogenicity avian influenza viruses (LPAIVs) that were phylogenetically distant from North American and Eurasian strains. The emergence of influenza A(H5N1) clade 2.3.4.4b virus in Eurasia marked a major evolutionary shift. The introduction of the HPAI H5N1 virus into the Americas in 2021 by migratory birds led to extensive reassortment with locally circulating LPAIVs (*2*), generating new genotypes that spread across the continent. By late 2022, the H5N1 virus reached South America, causing high mortality rates among seabirds and marine mammals (*3*,*4*). In 2023, H5N1 viruses were detected in wild birds and backyard chickens in Brazil (*3*). By 2025, new reassortant H5N1 variants had been reported in South America, including in Brazil (*5*). 

Beyond the emergence of novel genotypes, the continued expansion of HPAI viruses has been driven by broad host-range expansion and frequent interactions at the human–animal-environment interface. Those ecologic dynamics make mitigating the effects of HPAI viruses on wildlife, ecosystems, domestic animals, food security, and humans difficult (*6*). We report detection of a divergent HPAI H5N1 strain in 2 white-faced whistling ducks (*Dendrocygna viduata*) after reports of unusual avian deaths in Ibirapuera Central Park, São Paulo, Brazil. 

## The Study

In June 2025, we collected cloacal and tracheal swab samples from 2 dead white-faced whistling ducks (Appendix Figure 1) near the lake in Ibirapuera Park, São Paulo, Brazil (Figure 1). We detected AIV and H5N1 from the swab samples by using reverse transcription real-time PCR. We generated complete genome sequences (GenBank accession nos. PX062323–PX062330) (Appendix Table 1) directly from the original swab samples by using the Ion Torrent S5 Platform (Thermo Fisher Scientific, https://www.thermofisher.com). We classified genotypes by using GenoFlu (https://github.com/USDA-VS/GenoFLU). The hemagglutinin (HA), neuraminidase, polymerase acidic, and matrix protein (MP) segments were classified as Eurasian lineages, whereas the nonstructural protein segment was classified as a North American lineage. The remaining internal genes (polymerase basic 1, polymerase basic 2, and nucleoprotein) were unassigned (Appendix Tables 2, 3). We resolved the origins of the unassigned segments by using BLASTn and maximum likelihood (ML) analysis. The polymerase basic 2 and nucleoprotein sequences shared >94% nucleotide identity and clustered tightly with lineages from Colombia and the Brazilian Pantanal (*7*,^8^) (Figure 2). That topologic incongruence demonstrates reassortment with regional LPAIV gene pools. 

**Figure 1:**
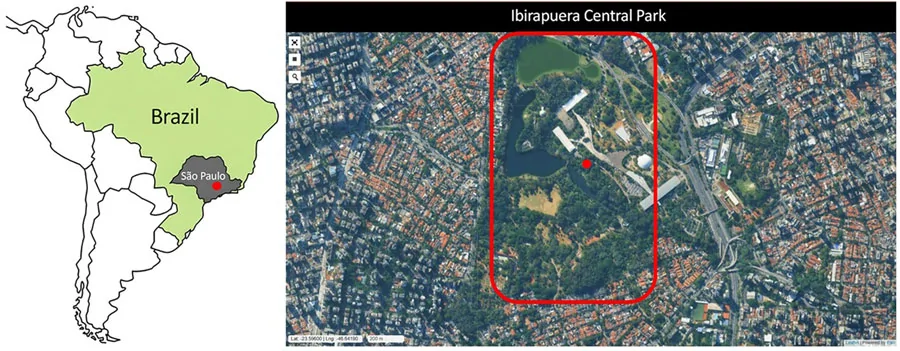
Location of Ibirapuera Central Park where waterfowl were found dead from divergent highly pathogenic avian influenza A(H5N1) clade 2.3.4.4b virus, São Paulo, Brazil, 2025. Map shows location in South America. Satellite image of park shows red outline of Ibirapuera Central Park, São Paulo, Brazil; red dots indicate the location where the 2 birds were found dead. Satellite image is from the catalog of images of the National Institute for Space Research (https://www.dgi.inpe.br/catalogo).

**Figure 2:**
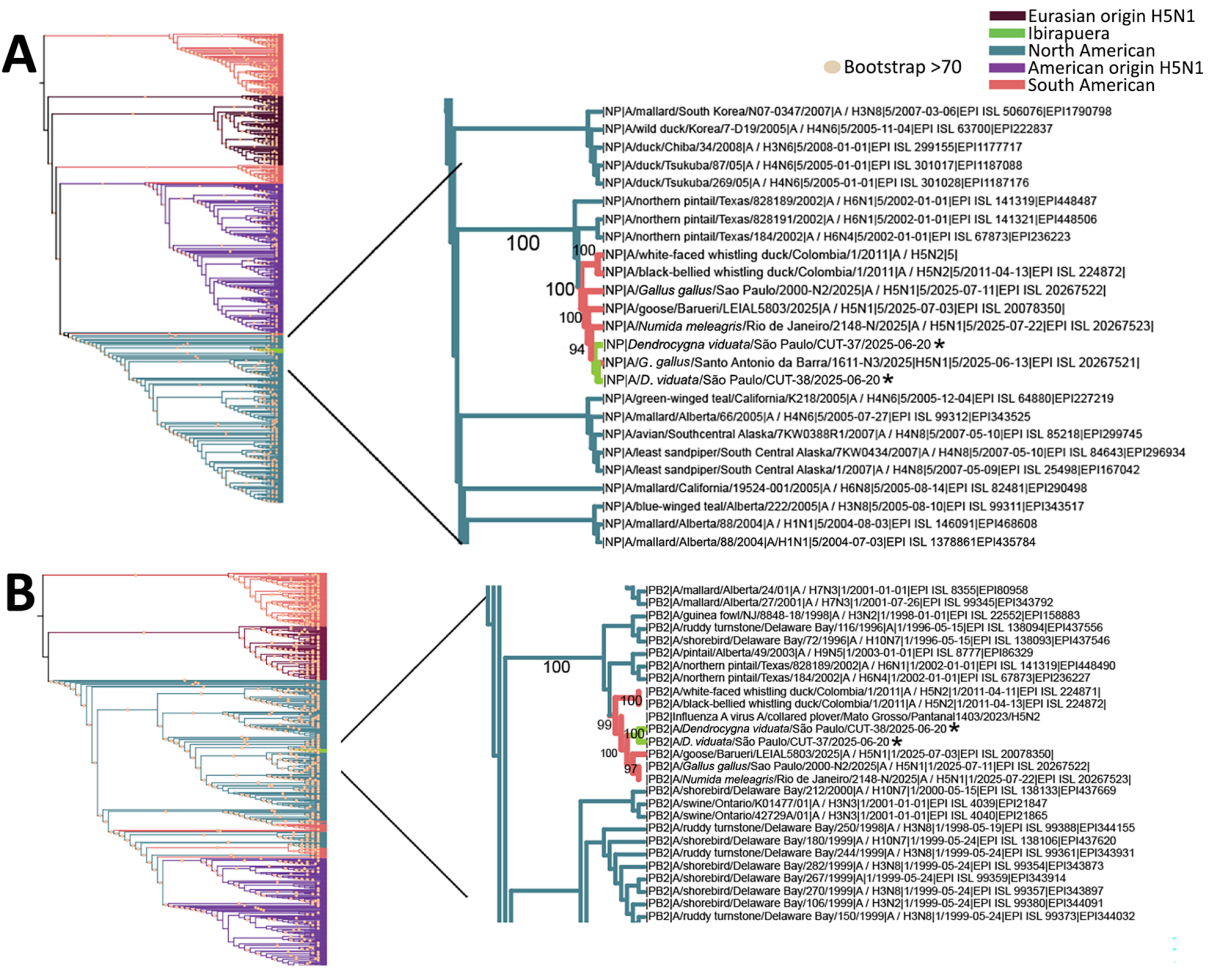
Maximum-likelihood phylogenies of NP and PB2 internal genes from divergent highly pathogenic avian influenza A(H5N1) clade 2.3.4.4b virus, São Paulo, Brazil, 2025. A) Alignment of NP internal gene sequences. B) Alignment of PB2 internal gene sequences. Asterisks (*) indicate sequences generated in this study. Numbers at major nodes indicate percent bootstrap values based on 1,000 replications. NP, nucleoprotein; PB2, polymerase basic 2.

Our phylogenetic analysis showed that the polymerase acidic and MP segments remain conserved within the Eurasian lineage, clustering with the clade 2.3.4.4b backbone, characteristic of the current panzootic (Appendix Figure 2). However, the nonstructural gene (North America lineage) clustered with AIVs established during the initial introduction of H5N1 viruses into the Americas (*2*) (Appendix Figure 3). Our genetic analyses of the HA gene sequences showed 97.9% nucleotide identity with the 2023 reference isolate from Brazil (GenBank accession no. OR269887.1) (*6*), and >98.6% nucleotide identity with recent North American strains (Appendix Table 3), suggesting a divergence from the original lineage established in Brazil. The maximum likelihood phylogenies we generated of the HA and neuraminidase genes also showed the sequence divergence (Figure 3; Appendix Figure 4). The isolates we sequenced form a monophyletic cluster among North American lineages (2022–2025) (*9*), suggesting a second introduction of the HPAI H5N1 virus in South America (*10**,**11*). 

**Figure 3:**
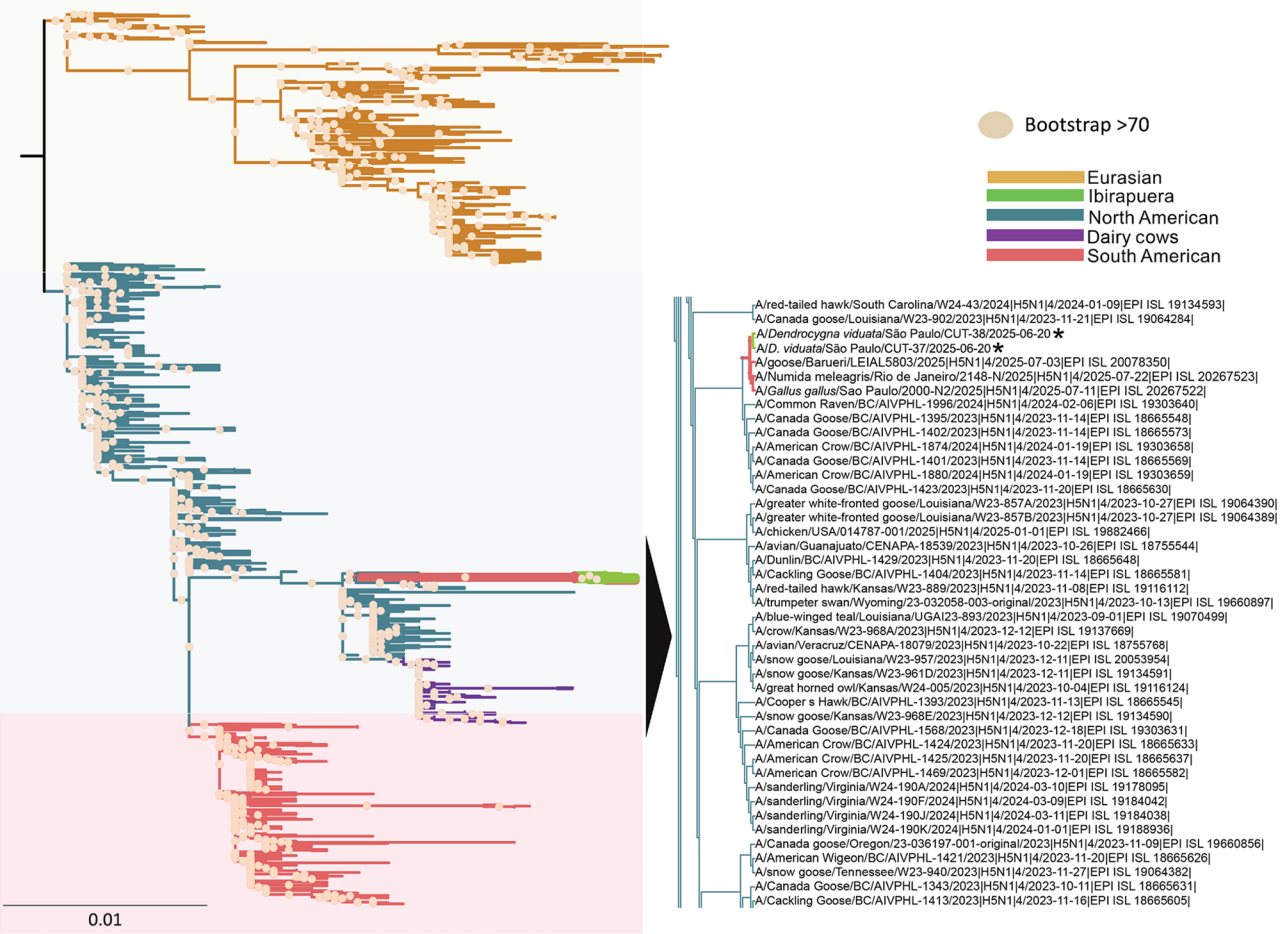
Phylogenetic tree of hemagglutinin gene from divergent highly pathogenic avian influenza A(H5N1) clade 2.3.4.4b virus, São Paulo, Brazil, 2025. Maximum-likelihood phylogenetic tree (left) showing alignment of global hemagglutinin gene sequences. Inset (right) shows closest related hemagglutinin gene sequences to those from this study denoted by asterisks (*). Scale bar indicates nucleotide substitutions per site.

We defined the timeline for the second introduction of the HPAI H5N1 virus into South America by using Bayesian temporal analysis. The estimated time to the most recent common ancestor (tMRCA) with North American H5 virus lineages was ≈mid-2023 (mean tMRCA May 2023; 95% highest posterior density March 2023–July 2023) (Figure 4). Our tMRCA estimates provide temporal support for the second H5N1 virus introduction from North America and subsequent circulation until it was detected in Ibirapuera Central Park. 

**Figure 4:**
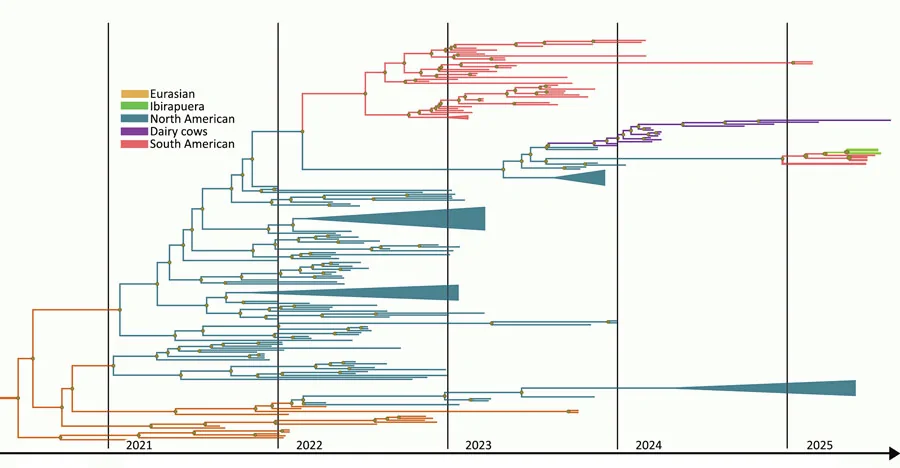
Time-scaled Bayesian maximum clade credibility tree of divergent highly pathogenic avian influenza A(H5N1) clade 2.3.4.4b virus, São Paulo, Brazil, 2025. The tree was inferred by using the Gaussian Markov Random Field Skyride prior model and includes 206 representative H5 clade 2.3.4.4b virus sequences from Eurasia and the Americas. Tan circle nodes indicate bootstrap >70.

## Conclusions

We identified a divergent reassortant HPAI H5N1 virus strain in white-faced whistling ducks from Ibirapuera Central Park, São Paulo, Brazil. As one of the city’s largest green, cultural, and recreational areas, attracting thousands of visitors each day (*12*), the park is a notable human–animal-environment interface. Following laboratory confirmation of H5N1, park authorities immediately implemented prevention, management, and isolation measures to reduce the risk for transmission.

Our phylogenetic and temporal analyses support a second introduction of HPAI H5N1virus into South America from North America, after reassortment with locally circulating LPAIVs in South America. Our findings suggest that reassortment likely occurred before the viruses dispersed through the interior regions of Brazil by regional migratory birds (*5*), although the precise movement dynamics of those waterfowl require further investigation. Similar exchanges have been described in other H5N1 genomes from South America (*5*,*11*), indicating ongoing genetic exchange between introduced HPAI viruses and regional AIV viruses, contributing to the increasing genomic diversity of H5N1 viruses circulating in South America. Together, those reassortment events position South America as a critical region for AIV surveillance. 

Our results highlight that HPAIV can be detected in a major urban park that harbors multiple animal species. The coexistence of resident waterfowl, migratory birds, synanthropic mammals, domestic animals, and abundant human activity creates an opportunity for cross-species transmission events. Our observations underscore the necessity of integrating systematic monitoring of urban wildlife into One Health surveillance frameworks to improve early detection and mitigate AIV risks.

Appendix Additional information about detection of divergent highly pathogenic avian influenza A(H5N1) clade 2.3.4.4b virus, São Paulo, Brazil, 2025.
