# Supplementary material for: Detection of Divergent Highly Pathogenic Avian Influenza A(H5N1) Clade 2.3.4.4b Virus, São Paulo, Brazil, 2025
Source: Emerg Infect Dis. 2026 Oct;32(10):1707–11. doi: 10.3201/eid3210.260723 (PMC13632867; doi:10.3201/eid3210.260723)
Supplement: Supplementary file 1 [file 26-0723-Techapp-s1.pdf]

Article DOI: https://doi.org/10.3201/eid3210.260723

EID cannot ensure accessibility for supplementary materials supplied by authors. Readers who have difficulty accessing supplementary content should contact the authors for assistance.

Detection of Divergent Highly Pathogenic Avian Influenza A(H5N1) Clade 2.3.4.4b Virus, São Paulo, Brazil, 2025

Appendix

1. Host Ecology and Viral Surveillance

The white-faced whistling duck (*Dendrocygna viduata*) has a wide pantropical distribution, spanning sub-Saharan Africa and much of South America, from Costa Rica to Uruguay. While it typically inhabits freshwater wetlands such as shallow lakes, marshes, and flooded rice paddies, the species also frequents urban parks and degraded water bodies. Unlike many waterfowl, *D. viduata* is not a long-distance migrant but is considered primarily resident, undertaking only moderate local or seasonal movements.

2. Materials and Methods

Sample Processing

Following reports of unusual avian mortality in Ibirapuera Central Park, São Paulo (23.588°S, 46.657°W) (Figure 1), in June 2025, two dead *Dendrocygna viduata* (white-faced whistling ducks) were recovered (Appendix Figure 1). Cloacal and tracheal swabs were collected and transported on dry ice to the Laboratory for Research on Emerging Viruses (LPVE-ICB-USP) under biosafety level 3 conditions. Clinical specimens were screened for Influenza A through qPCR, targeting the matrix (MP) gene and specific qPCR for HPAI H5. Samples were isolated in controlled embryo chicken eggs after initial detection and stored at LPVE-ICB-USP. Immediate notification was made to the authorities and the Ministry of Agriculture (MAPA), with samples sent to the reference laboratory for subsequent confirmation. Immediately after the first detections, prevention and control measures were implemented by the park’s veterinary and

biology teams. These included isolating the affected area, restricting public access, safely removing carcasses, and enhancing biosecurity to prevent contact between wildlife and visitors. The Agricultural Defense Agency informed through public communication that measures were adopted to inform visitors and discourage contact with sick or dead birds (Official accession:https://www.defesa.agricultura.sp.gov.br/noticias/2025/influenza-aviaria-apos-novo-caso-positivo-em-ave-silvestre-defesa-agropecuaria-reforca-atividades-educativas-para-conscientizar-a-populacao,2309.html). Other resident birds were tested, and no additional mortality events involving mammals or other bird species were documented with H5N1 during the investigation period. The health department was notified to monitor park workers for any influenza-like symptoms, and no symptoms were reported among them.

To better emphasize the collaboration among municipal wildlife authorities (CeMaCAS), the Municipal Secretariat for Green and Environment, academic laboratories, and public health institutions. This coordinated response illustrates a practical One Health framework integrating wildlife surveillance, laboratory diagnostics, environmental management, and public health risk communication. We believe that these additions considerably strengthen the One Health component of the manuscript.

Full Genome Amplification and Sequencing

To prepare for whole genome sequencing (WGS), genome amplification of all IAV segments was achieved through a PCR, using primers described by Zhou *et al* (2009). The reaction was carried out using a one-step PCR with the SuperScript IV system direct from clinical specimens. WGS was carried out under the Ion Torrent S5 Platform. The construction of the libraries followed the protocol of the kit Ion Xpress Plus Fragment Library Kit (Ion Torrent). Raw FASTQ files were trimmed with Cutadapt and assembled using SPAdes v3.15.5 with the “Iontorrent” parameter. AIV genome segments were identified by BLAST, and trimmed reads were mapped to the corresponding assembled viral genomes using BWA to generate coverage metrics (Appendix Table 1).

Genome Identification

To evaluate reassortment, we looked for overall tree topologies, and the clustering patterns of closely related sequences were analyzed in conjunction with automated genotyping using the GenoFLU tool (https://github.com/USDA-VS/GenoFLU) (Appendix Table 2**)**. For

gene segments that were not assigned to a definitive genotype by the automated tool ("unassigned"), their identification was inferred through manual inspection of the ML phylogenies, and clustering with lineages supported by high bootstrap reliability values (>90) was considered as evidence of reassortment events. To assess nucleotide similarity of all segments, we identified the most related genomes through searches in the NCBI and GISAID databases. Additionally, pairwise alignments of the HA gene using Geneious Prime were made, comparing our isolates against reference sequences from 2021 to 2025 (Appendix Table 3).

Dataset Criteria and Phylogenetic Inference

To perform phylogenetic inferences, datasets for Maximum Likelihood (ML) and Bayesian inference were compiled. For ML, the dataset comprised the 500 closest sequences identified in the GISAID database. Additionally, we incorporated well-characterized sequences of the H5N1 clade 2.3.4.4b from Eurasia, North America, and South America spanning from 2020 to 2025. Multiple sequence alignments were conducted using MAFFT. Phylogenetic reconstruction was performed using ML estimation (IQ-TREE 2, substitution model defined for each segment with ModelFinder) with ultrafast bootstrap support. ML trees were visualized using iTOL, and figures for papers were edited using Affinity software (Appendix Figures 2–4). To reconstruct the temporal evolutionary history and estimate the time to the most recent common ancestor (tMRCA) of the HA gene, we performed a Bayesian phylogenetic analysis using the BEAST v10.5 software package (Figure 4). From the prior ML dataset, a subsampled dataset of 206 sequences was compiled for the Bayesian analysis. Temporal signal in the dataset was assessed using TempEst v1.5.3 through root-to-tip regression analysis (Appendix Figures 5,6). Bayesian inference was conducted under an uncorrelated relaxed molecular clock model with a GMRF Bayesian Skyride coalescent demographic model. Two independent Markov chain Monte Carlo (MCMC) chains were run for 100 million states. Convergence and effective sample sizes (ESS >200) were assessed using Tracer. Log files were combined using LogCombiner v10.5 after removal of 10% of burn-in, and maximum clade credibility (MCC) trees were summarized using TreeAnnotator v10.5. Phylogenetic trees were visualized in FigTree, and final tree figures were edited using Affinity Studio software.

Appendix Table 1. Genome metrics of the obtained segments.

Segment	Mean coverage (CUT37)	Mean coverage (CUT38)
PB2	2632.99	1631.11
PB1	486.36	419.98
PA	1238.36	822.70
HA	5801.48	3884.90
NP	9910.86	5280.70
NA	6217.97	3970.15
MP	18429.40	9644.56
NS	18422.30	9416.06

Total reads: CUT37 = 443446; CUT38 = 222646. GenBank accession: CUT37 = PX062323 - PX062330; CUT38 = PX062331.1 - PX062338.1.

Appendix Table 2. Genetic characterization of H5N1 virus – CUT37/38. Segment genotypes and BLAST hits showing gene origins.

Segment	Genotype	Top BLAST Hit	% nt Identity	Gene Segment Origin
PB2	N.A	A/black-bellied whistling duck/Colombia/1/2011(H5N2)	>94.2%	South American
PB1	N.A	A/Mallard/Illinois/170S2601/2017(H4N6)	>97.5%	North American
PA	ea1	A/Aves/Guanajuato/CPA-18539–23/2023(H5N1)	>99.5%	North American
HA	ea1	A/Aves/Guanajuato/CPA-18539–23/2023(H5N1)	>98.9%	North American
NP	N.A	A/black-bellied whistling duck/Colombia/1/2011 (H5N2)	94.9%	South American
NA	ea1	(A/blue-winged teal/Louisiana/UGAI23–893/2023(H5N1)	>98.9%	North American
MP	ea1	(A/greater white-fronted goose/Louisiana/2023 (H5N1)	>99.0%	North American
NS	am1.1	(A/red-tailed hawk/South Carolina/W24–43/2024(H5N1)	>98.0%	North American

N.A- unassigned, ea1- and am1.1- assigned e classified

Appendix Table 3. Nucleotide similarity of H5N1 virus. HA gene nucleotide similarity across H5N1 strains.

Strain (H5)	Nucleotide similarity
A/Mallard_Duck/Netherlands/28/2022	96.5%
A/Canada_Goose/BC/AIVPHL-844/2022	97.7%
A/Thalasseus_maximus/Sao_Paulo/2023	97.9%
A/Green_Winged_Teal/BC/AIVPHL-2045/2024	98.7%
A/feline/Texas/24–029329–01/2024	98.7%
A/dairy_cow/USA/007549–125/2025	98.6%
A/Aves/Guanajuato/CPA-18539–23/2023	98.9%
HA | A/goose/Barueri/LEIAL5803/2025	99.6%

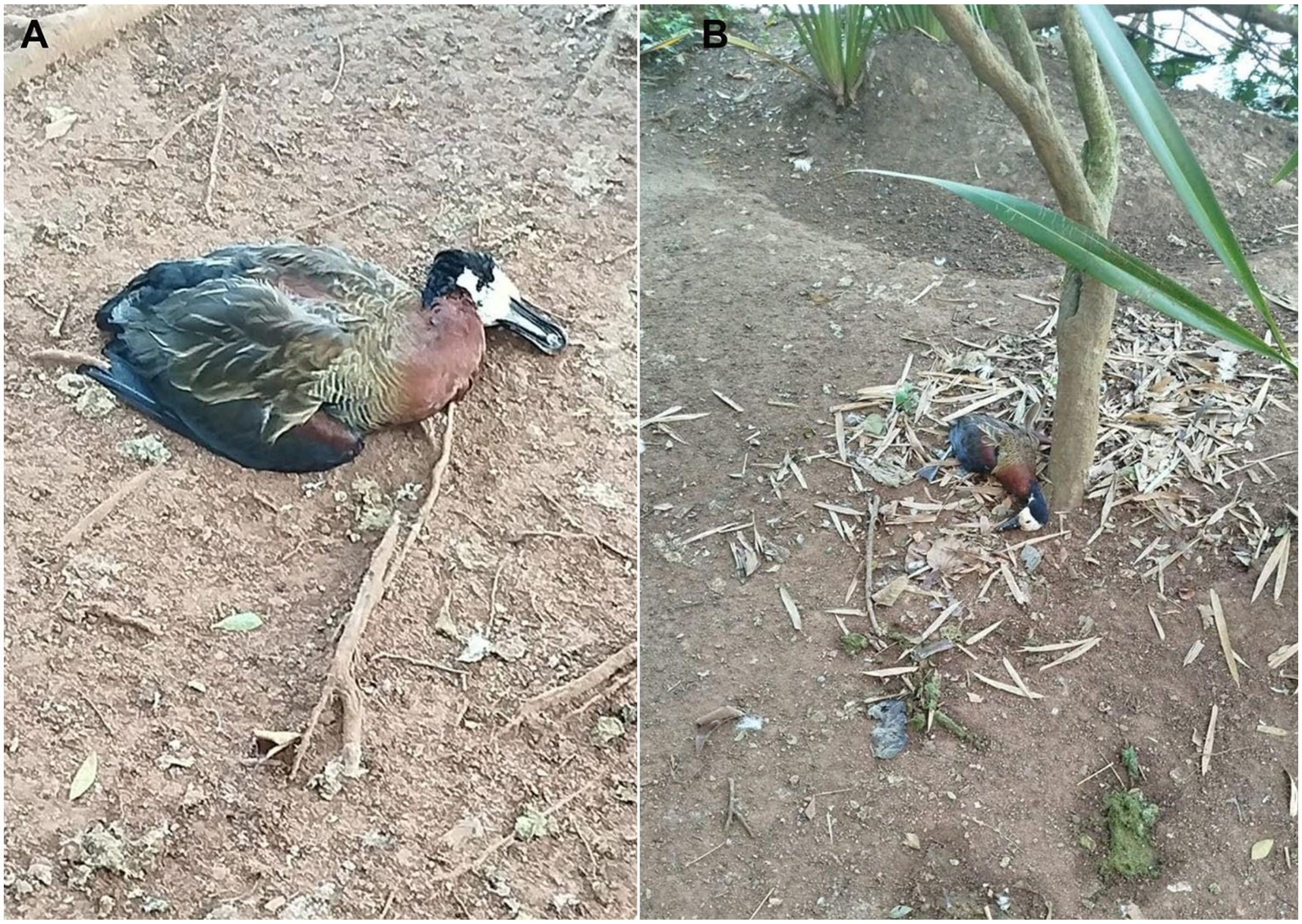

Appendix Figure 1. Dead White-faced Whistling Duck individuals recorded in *Ibirapuera* Central Park, illustrating field conditions at the sampling site.

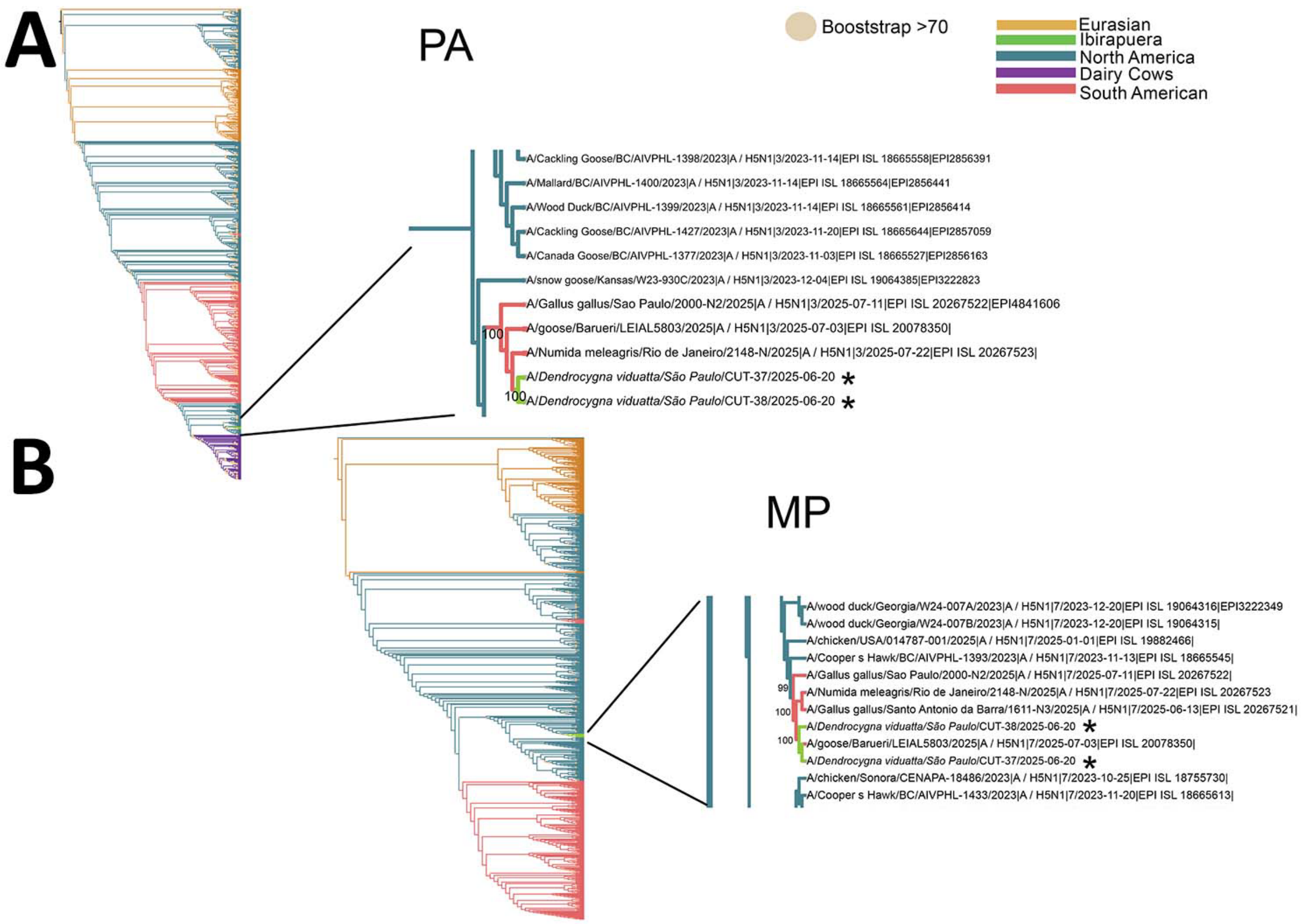

Appendix Figure 2. Maximum likelihood phylogenies of the PA and MP internal genes. Ibirapuera H5N1 strains (green) cluster with South and North American LPAI sequences. Bootstrap values are indicated by light orange dots on complete trees and numerically in close-ups.

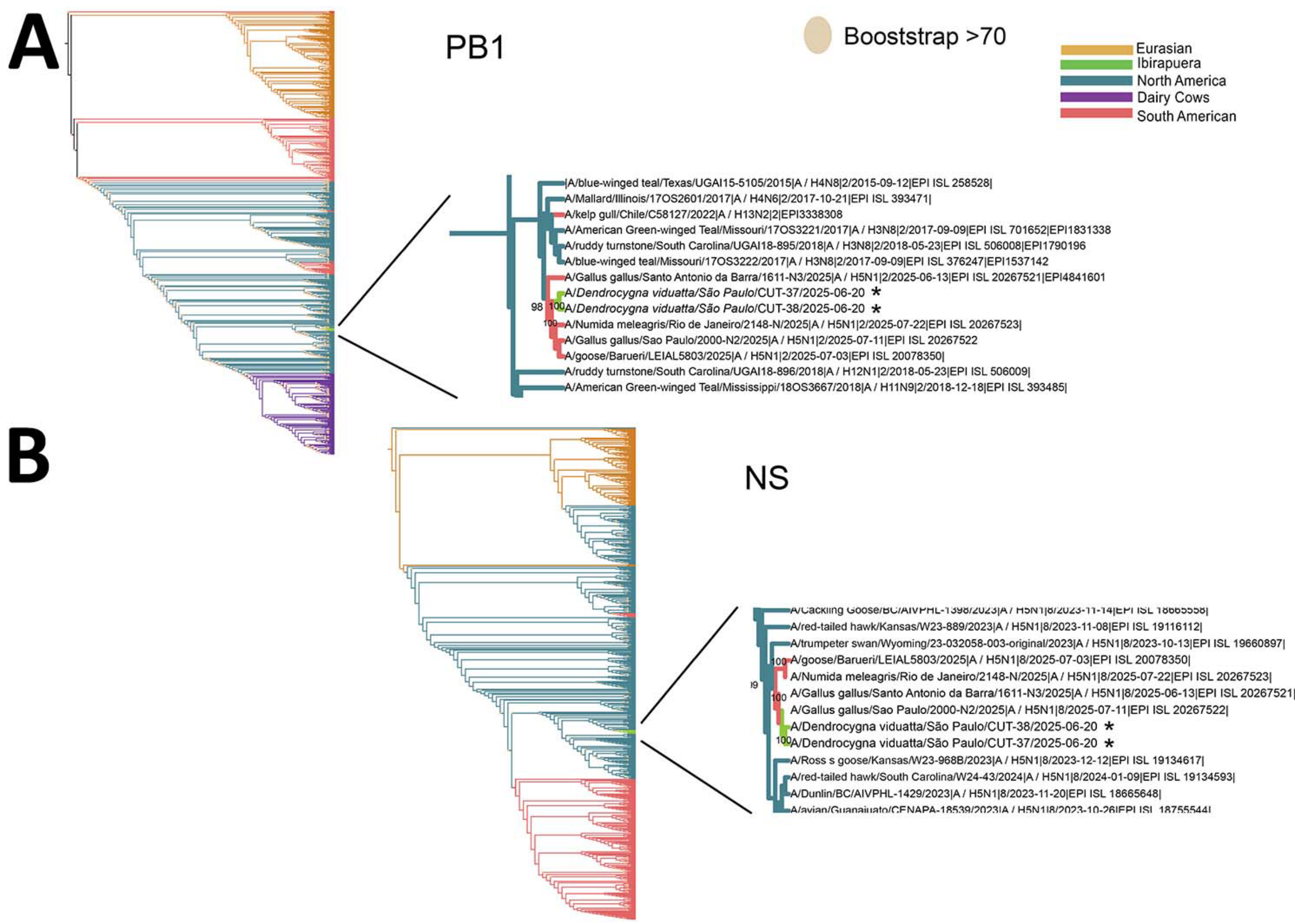

Appendix Figure 3. Maximum likelihood phylogenies of the PB1 and NS internal genes. Ibirapuera H5N1 strains (green) cluster with South and North American LPAI sequences. Bootstrap values are indicated by light orange dots on complete trees and numerically in close-ups.

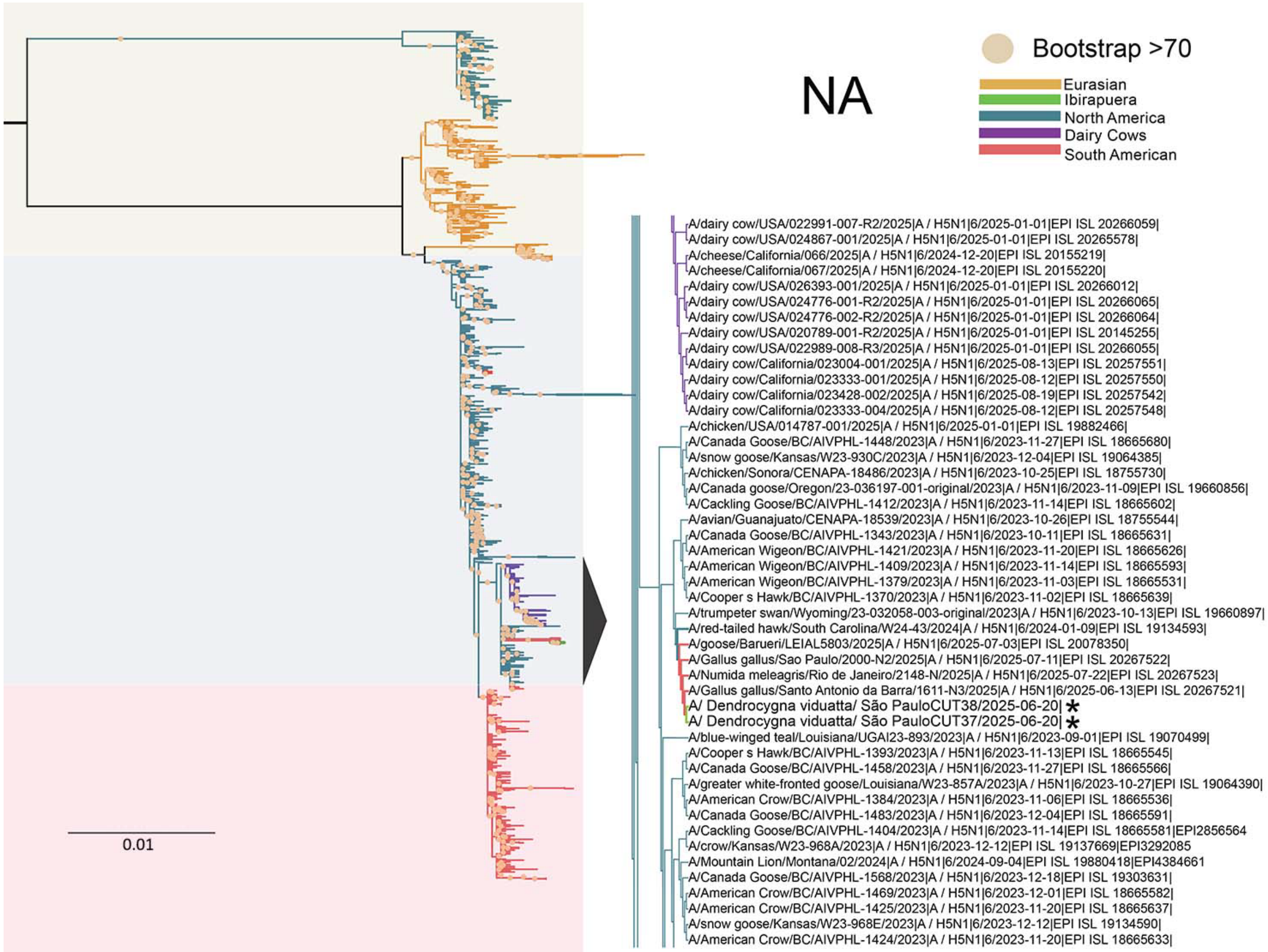

Appendix Figure 4. Maximum Likelihood phylogenetic tree of the H5N1 Neuraminidase (NA) gene. Like the HA gene, the Ibirapuera isolates (highlighted in green) form a robust monophyletic cluster with Brazilian sequences of 2025, distinct from the initial 2022–2024 South American lineage (red). This clade groups within the recent North American diversity (blue).

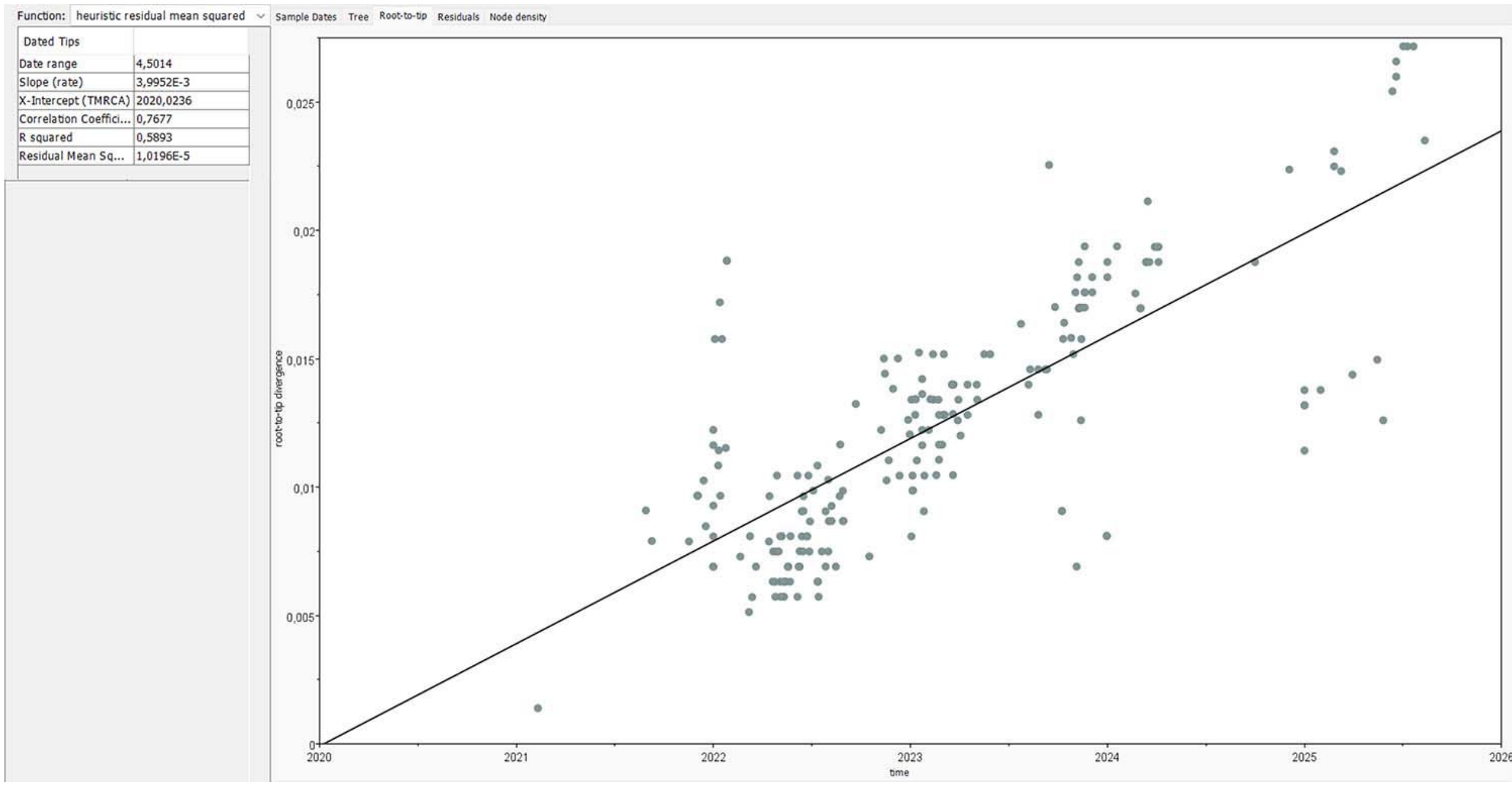

Appendix Figure 5. Root-to-tip divergence plotted against sampling time using TempEst. The positive correlation (R^2 = 0.7677) indicates a strong temporal signal in the dataset, supporting subsequent Bayesian phylogenetic inference. The regression estimates an evolutionary rate of ≈3.99 × 10^{-3} substitutions per site per year and a time to the most recent common ancestor (tMRCA) of 2020.02.

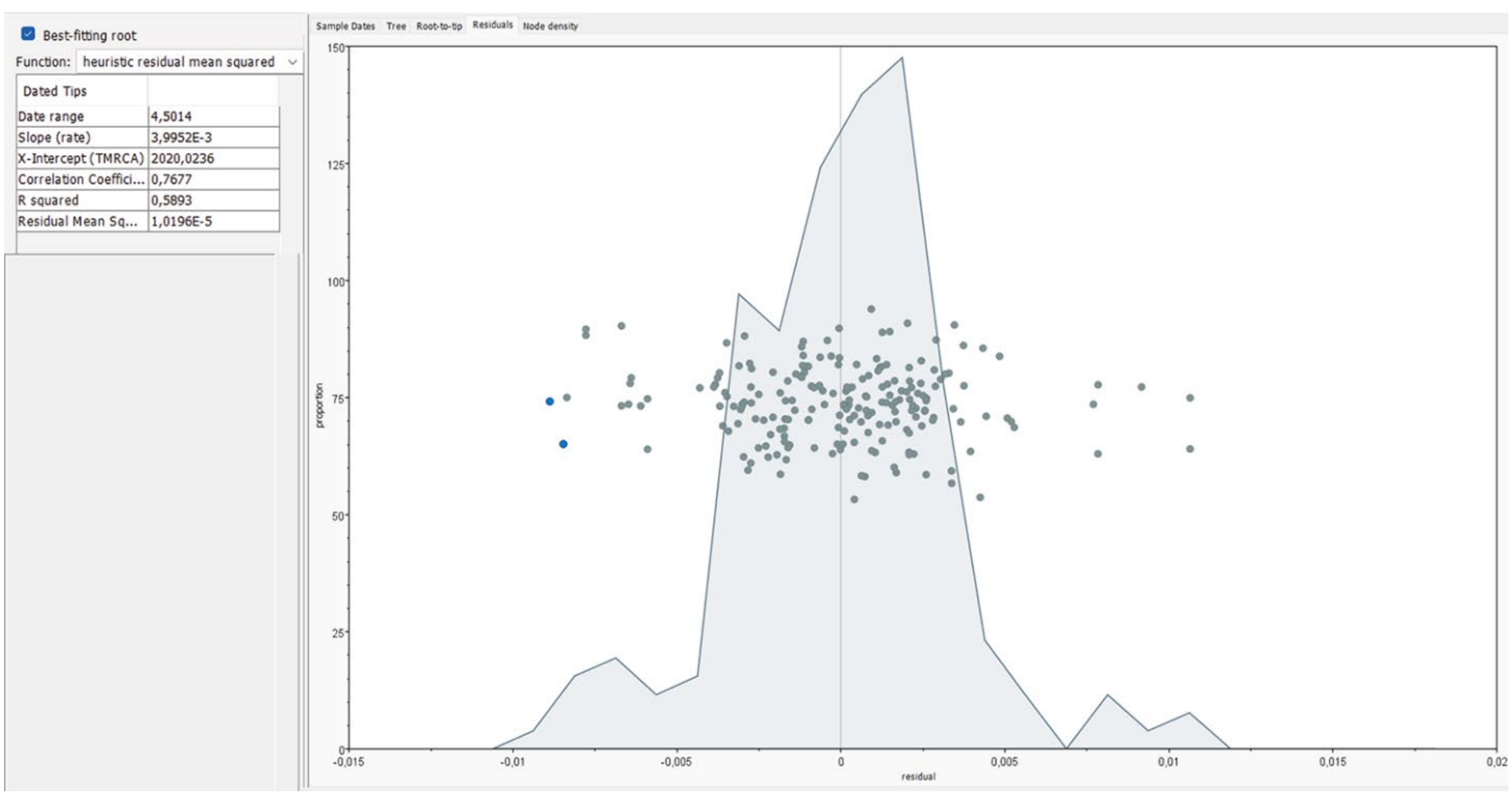

Appendix Figure 6. Residual plot from the root-to-tip regression (Tempest). The distribution of residuals around zero evaluates the fit of the molecular clock model and helps identify potential sequence outliers.
